# Prevalence of HIV Antiretroviral Drug Resistance and Its Impacts on HIV-1 Virological Failures in Jiangsu, China: A Cross-Sectional Study

**DOI:** 10.1155/2016/1752437

**Published:** 2016-10-11

**Authors:** Ying Zhou, Jing Lu, Jinge Wang, Hongjing Yan, Jianjun Li, Xiaoqin Xu, Zhi Zhang, Tao Qiu, Ping Ding, Gengfeng Fu, Xiping Huan, Haiyang Hu

**Affiliations:** ^1^Jiangsu Provincial Center for Disease Prevention and Control, Nanjing, China; ^2^College of Agriculture, Nanjing Agriculture University, Nanjing, China

## Abstract

Antiretroviral therapy (ART) has been shown to improve survival of patients with Human Immunodeficiency Virus (HIV) infection and to reduce HIV-1 transmission. Therefore, the Chinese central government initiated a national program to provide ART free of charge to HIV-1 patients. We conducted a cross-sectional survey in Jiangsu province to determine the level of drug resistance (DR) in HIV-1 infected patients and the correlates of DR in virological failures in 2012. Approximately 10.4% of the HIV-1 patients in the study experienced virological failure after one year of ART and were divided into drug sensitive and drug resistant groups based on genotype determination. The viral loads (VLs) in the drug resistant group were significantly lower than the drug sensitive group. There were two independent predictors of virological failure: male gender and increasing duration of treatment. The primary mutations observed in the study were against nucleoside reverse transcriptase inhibitors (NRTIs) which were M184V (79.45%) and K103N (33.70%) in nonnucleoside reverse transcriptase inhibitors (NNRTIs). The overall rate of DR in Jiangsu province is still relatively low among treated patients. However, close monitoring of drug resistance in male patients in the early stages of treatment is vital to maintaining and increasing the benefits of HIV ART achieved to date.

## 1. Introduction

During 1996–2012, it was estimated that antiretroviral therapy (ART) prevented approximately 6.6 million deaths due to Acquired Immunodeficiency Syndrome (AIDS) worldwide, including 5.5 million deaths in low- and middle-income countries [1]. In addition, ART has been shown to reduce the risk of Human Immunodeficiency Virus (HIV) transmission [2–4]. Given the potential benefits of effective ART treatment, the Chinese central government launched the National Free ART Program in 2002. The program has expanded rapidly since 2004 [5], and by September 2013, the National Free ART Program has cumulatively treated approximately 260,000 patients [6]. It is estimated that during 2002 to 2009 the program reduced AIDS associated mortality from 39.3 to 14.2 cases per 100 infected persons [7]. The promising early results from the National Free ART Program have resulted in strong governmental supports for continuing to improve and expand the program. The treatment guidelines for the Chinese National Free ART Program have been carried out with zidovudine (AZT)/stavudine (D4T) + didanosine (DDI) + nevirapine (NVP) as the regimens in 2003 [8] and switched to AZT/D4T + 3TC + EFV/NVP as first-line therapy regimens in 2005. Because of the side effect of D4T, the first-line regimens were switched again to TDF + 3TC + NVP/EFV in 2012 lasting until now to remain current with the World Health Organization (WHO) ART guidelines, including the latest 2013 WHO guidelines [9, 10].

While increasing the availability of ART has improved the survival rates and quality of life for HIV/AIDS patients, resistance to antiretroviral drugs can undermine the success of ART programs [11]. A high number of ART resistant HIV-1 strains have emerged after ART in resource-limited countries and substantially offset the benefits from treatment programs [12, 13]. Similarly, a trend has been observed in China. There was a concomitant increase in the number of HIV patients with ART resistance during the ramp up of the National Free ART Program [14, 15]. To decrease the diminishing efficacy of the free antiretroviral treatment, China initiated the switch to second-line antiretroviral therapy for patients with failure of first-line treatment in 2009 [8]. However, second-line antiretroviral therapy is the last choice in China in HIV-1 treatment because of the drug deficiency.

Jiangsu is a representative province in eastern China for societal characteristic and the prevalence of HIV-1 disease [16]. Free ART was implemented in Jiangsu in 2005 as part of the Chinese National Free ART Program. The number of new reporting cases of HIV-1 rapidly increased from 78 in 2005 to 3841 in 2012. However, there was no comprehensive effort made to collect data about these patients, including basic information such as viral loads (VLs), and the prevalence of drug resistant HIV-1, until after they had been receiving ART for at least one year. In this study, we have undertaken the first comprehensive examination of the HIV-1 patients in Jiangsu province in 2012 to document the rates of DR in virological failures and correlates of DR in HIV-1 virological failures receiving ART for at least 12 months. The results of this study will help provide a baseline evaluation of the Chinese HIV-1 treatment program and may help inform evidence-based decision making for clinical care of HIV-1 patients in China.

## 2. Methods

### 2.1. Ethical Statement

This was approved by the Ethics Committee of Jiangsu Provincial Center for Disease Prevention and Control. Signed informed consent was obtained from each of the participants prior to the interviews and blood collection. Each participant was free to decline to participate or withdraw from the study at any point in time. All the methods (not just laboratory experiments) were carried out in accordance with the relevant guidelines, including any relevant details.

### 2.2. Study Design

We conducted a cross-sectional survey of all patients who were currently on ART for at least 12 months from January 1 to December 31, 2012, in Jiangsu province. Laboratory data and demographic information were collected to evaluate HIV drug resistance.

### 2.3. Sample Selection and Information Collection

All the laboratory experiments were carried out in accordance with approved guidelines. According to the guidelines of the National Free ART Handbook (version 3, 2012) [11], HIV-1 cases whose CD4^+^ T-cell count ≤350 cells/mm^3^ are eligible for ART. Free CD4^+^ T-cell counts tests were conducted twice each year for all treated patients and free VLs testing was conducted once per year for patients who received ART for more than 12 months. Patients with VLs >1,000 copies/mL after 12 months of treatment were defined as virological failures and selected for further drug resistance genotyping test according to this ART Handbook too. The presence of any drug resistant mutation whatever it is in NRTIs, NNRTIs, or PIs is defined as drug resistance in this study. The inclusion criteria for this study were as follows: (1) patients had to be receiving ART for more than 12 months; (2) they had to have VLs testing results; and (3) there was access to serum samples at the Center of Jiangsu Provincial HIV/AIDS confirmatory laboratory, Jiangsu Provincial Center for Disease Prevention and Control (JSCDC). We allowed 16 cases who had been treated for between 11 and 12 months to participate in the study. All of the laboratory results and patient demographic information related to ART were entered into the web-based national ART information system. Demographic information including age, gender, and treatment duration and other relevant information required for the HIV drug resistance analysis were collected from the China Information System for HIV Control and Prevention.

### 2.4. Laboratory Measures

VLs and drug resistance testing were conducted at the provincial HIV/AIDS confirmatory laboratory. The VLs were quantified using the COBAS Ampliprep TaqMan 96 (Roche) and COBAS TaqMan HIV-1 Test v2.0 kit. HIV-1 RNA was extracted from the plasma using the QIAamp Viral RNA Mini Kit. Both VLs testing and HIV-1 RNA extraction were performed using the residual blood drawn for the CD4^+^T-cell testing. The residual blood was stored at −80°C until it was analysed.

Nested-PCR was used to amplify the reverse transcriptase gene (codons 1–230) and protease gene (codons 1–99) from the virus isolated from the plasma [17]. The PCR products were sent to Company TaKaRa for sequencing and the resulting sequences were spliced together using the software Chromaspro. Interpretation and analysis of the sequences generated were undertaken using the Stanford University HIV Drug Resistance Database (http://hivdb.stanford.edu/).

### 2.5. Statistical Analysis

VLs were analysed with *F* test to compare variances using the Graphpad Prism4 software (Graphpad Software, Inc.) and *p* < 0.05 was considered as significant. The factors that could have been related to drug resistance were collected from the China Information System for HIV Control and Prevention. Univariate associations were evaluated using the chi-square test for categorized variables and the Student *t*-test for continuous variables and the two-sided test to estimate the level of significance. The selected variables of *p* < 0.1 in univariate analysis were entered in the logistic regression model to determine level of significance with SPSS version 20.0 (IBM).

## 3. Results

### 3.1. Patient Demographics

At the end of 2012, the VLs had been determined for 2,223 HIV-1 patients who had received ART for at least one year. Of these, 232 patients had VLs >1,000 copies/mL and were classified as virologic failures who needed to be further tested for drug resistance. The overall rate of virologic failure was 10.44% (232/2223). Of 232 cases of virological failure, 196 plasma samples were successfully taken to test for drug resistance by genotyping. Of these, 101 were considered drug resistant and 95 were drug sensitive. Therefore, the overall rate of DR in Jiangsu HIV-1 patients sample was 4.54% (101/2223).

Next, we compared the VLs between drug sensitive and resistant groups. There was a statistically significant difference in the mean VLs between the drug sensitive (303,500 ± 201700 copies/mL) and the drug resistant (75,860 ± 15080 copies/mL; *p* < 0.05* F* test to compare variances).

### 3.2. Factors Associated with Drug Resistance

The demographic characteristics of HIV-1 treated patients with virological failure for whom plasma samples could be obtained for resistance testing are shown in Table 1. Patients were most commonly male, 30–50 years old, and married, had contracted HIV via the heterosexual route, were classified as clinical stage I HIV by the WHO, had been treated for 1-2 years, and were frequently treated with AZT/D4T + 3TC + NVP/EFV (NRTIs + NNRTIs combination). A logistic regression analysis was conducted to identify independent predictors of drug resistance in this population. Patients who were female (RR = 0.362, 95% CI: 0.155–0.844) were at lower risk of ART resistance. In addition, by comparing the relative risk of drug resistance between patients treated for 1-2 years (RR = 13.616, 95% CI: 1.715–108.109), 2-3 years (RR = 19.556, 95% CI: 2.278–167.857), and more than 3 years (RR = 50.579, 95% CI: 4.855–526.891) we concluded that there was a significantly greater possibility of HIV DR emerging with longer treatment duration (Table 2).

**Figure 1:**
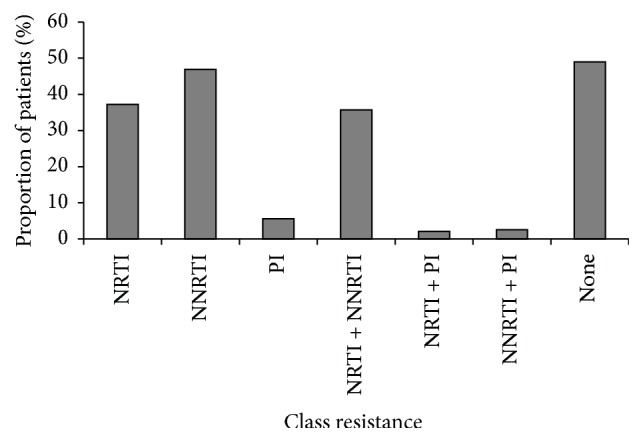
The percentage of patients with specific antiviral mutations. Based on the genotype results, the drug resistant strains were divided into nucleoside reverse transcriptase inhibitor (NRTI); nonnucleoside reverse transcriptase inhibitor (NNRTI); and protease inhibitor (PI) resistant strains using the Stanford website. The percentage of patients with NRTI, NNRTI, or PI resistant strains is shown. The denominator in all cases is 196 patients, all those who experienced virological failure and had serum samples available. The numerators for each column were NRTI = 73 (including multiple drug resistant strains); NNRTI = 92 (including multiple drug resistant strains); PI = 11 (including multiple drug resistant strains); NRTI + NNRTI = 70; PI + NRTI = 4; and PI + NNRTI = 5. In addition, 95 strains had none of the drug resistance mutations.

To better understand the types of DR observed in Jiangsu, we further subdivided the drug resistant cases by resistance to nucleoside reverse transcriptase inhibitors (NRTIs), nonnucleoside reverse transcriptase inhibitors (NNRTIs), or protease inhibitors (PIs) based on mutations in the viral genome. The highest frequency drug resistance mutations were NNRTIs (46.94%), followed by NRTIs (37.25%), and PIs (5.61%). These results suggested that NNRTIs drug use caused the most severe resistance in Jiangsu. We then compared the frequency of multidrug resistance (NRTIs + NNRTIs, NRTIs + PIs, and NNRTIs + PIs). The combination with the highest frequency of multidrug resistance was the NRTIs and NNRTIs (35.17%) combination (Figure 1).

### 3.3. DR Mutations Profiles

**Figure 2:**
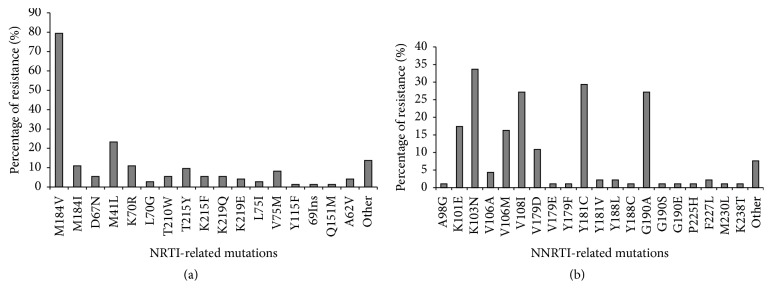
The frequencies of resistant mutations associated with NRTI. The results of these mutations presented here are all from the patients' sequence alignment on Stanford University HIV Drug Resistance Database. The percentages of mutation were showed in the figures. (a) The denominator in these cases is the number of patients who get any NRTI mutations (*n* = 73). M184V (*n* = 58), followed by M41L (*n* = 17), M184I (*n* = 8), and K70R (*n* = 8) in sequence. (b) The denominator in these cases is the number of patients who get any NNRTI mutations (*n* = 92). The most numerous mutation in NNRTI is K103N (*n* = 31), followed by Y181C (*n* = 27), G190A (*n* = 25), and V108I (*n* = 25).

The most common mutations associated with drug resistance in NRTIs were M184V (79.45%), M41L (23.29%), M184I (10.96%), and K70R (10.96%) (Figure 2(a)). K103N, Y181C, G190A, and V108I were the most prevalent mutations associated with NNRTIs resistance and the frequencies were 33.70%, 29.35%, 27.17%, and 27.17%, respectively (Figure 2(b)). The most often combination of NRTIs mutations is M184V + M41L, and its frequency is 6.63%; however, the pattern of NNRTIs mutation combination is an even distribution. 69.30% of patients (70/101) harbored resistance to both NRTIs and NNRTIs. Four PI-associated mutations were observed in 11 individuals in this study whithout phenotypic resistance to PI drugs.

## 4. Discussion

Jiangsu is the largest province in eastern China; therefore, incomplete data about the efficacy of the National Free ART Program in Jiangsu affects the integrity of the data for China as a whole. Here, we have analysed the prevalence of DR in Jiangsu and characterised the factors associated with drug resistance in virological failure in 2012. This particular year was selected because there were a large number of samples available and there was a more complete collection of data for each case available. This study provides a baseline for further study on the efficacy of the ART program. We found that failure occurred overall in about 10.44% of cases and that the overall rate of DR was about 4.54%. The prevalence of HIV DR is low compared to the WHO estimate suggesting the emergence of drug resistance in <30% of HIV-1 infected patients with virological failure [18]. Furthermore, this figure indicates that the ART treatment regimens in Jiangsu are efficacious. Plasma VLs and blood CD4 cell counts are most widely used to monitor the success of antiretroviral therapy. Surprisingly, in those cases where virological failure occurred, we observed higher VLs results in patients with the drug sensitive virus. It is reported that the replication capacity of HIV drug resistant strains would be decreased during the course of viral RNA synthesis in contrast to the wild type HIV virus [19]. In addition, it has been shown in other studies that the risk of selection of resistance peaks around 10,000 copies/mL [20]. All these findings above are in accordance with our results of VLs in drug-resistant group which is lower than that in drug-susceptible group. But there is no evidence demonstrating that VLs show correlation with drug resistance incidence [21].

Mutations associated with DR were found in approximately half (51.5%) of the plasma samples from patients with virological failure. This estimation is similar to nationwide rates of HIV drug resistance among patients with treatment failure (57%) [22]. Also consistent with previous studies, NNRTIs, NRTIs, and the NRTIs + NNRTIs combination regimens showed high drug resistance [22]. The majority of patients enrolled in our study received treatment with a NRTIs + NNRTIs combination regimen for 1-2 years. In fact, studies showed that drug resistance most often emerged in the first 6–12 months after ART initiation [23]. However, it is hard to practice this surveillance guideline in resource-limited countries. Therefore, the WHO has developed comprehensive drug resistance surveillance and monitoring strategy based on public health principles [24], to address concerns about emergence of drug resistance, and developed early warning indicators (EWI) for use in resource-limited settings to limit the development and spread of preventable drug resistance [25]. Therefore, it would likely benefit the Chinese program to introduce the WHO EWI to limit the possibility of further drug resistance emerging in Jiangsu. The high rate of resistance to the first-line regimen also suggests that we should be aware of the possibility of transmitted drug resistance (TDR) and test for drug resistance in treatment-naïve HIV-1 infected patients.

The resistance mutations identified in this study are not surprising for a developing country treatment program based on NRTIs and NNRTIs, with second-line PI regimens not yet scaled up. M184V could cause high-level resistance to 3TC and low-level resistance to ddI. M41L belongs to TAMs (thymidine analogue-associated mutations) that were selected by AZT and D4T. K103N, Y181C were the most prevalent mutations associated with NNRTI resistance in our study. K103N causes high-level resistance to NVP and EFV and Y181C can cause high-level resistance to NVP and intermediate resistance to EFV. All the mutations identified are in accordance with our first-line therapy regimen. Of concern, in 101ART treated patients with drug resistance mutations, 69.30% harbored resistance to both NRTI and NNRTI, much higher than the incidence around 50% in other studies in China [26, 27]. The number of incidences in Africa is around 60% and it is much lower in North America or in Western Europe, which are both approximately 20% [28]. This number of double resistance to NRTIs and NNRTIs in our study was particularly high partly due to the sustaining, permanent ART regimen without new drugs alternative.

Logistic regression analysis showed that gender and treatment time were associated with DR. The findings show that male gender and long-term ART predicted drug resistance are similar to previous studies in the Chinese literature [29, 30]. In Jiangsu province, the main route of HIV transmission was through heterosexual transmission; however, homosexual transmission has surpassed heterosexual transmission in recent years [31]. In our study, 34.7% of male patients with virological failure were men who have sex with men (MSM). Compared with other demographic groups infected with HIV, MSM tend to have higher drug resistance rates and worse treatment outcomes [32, 33]. Thus, additional monitoring and further assessments of the drug resistance profile in this population are required to consolidate the efforts for ART treatment.

Our study is limited by several caveats. First, of the 232 patients that were virological failures, only 196 were successfully sequenced, meaning 15.5% (36/232) of the samples were missing for sequences analysis, and the estimated prevalence of DR therefore may not be accurate and credible. Second, our conclusions only reflect the status of DR in 2012, additional studies are necessary to fully evaluate the consequences of the National Free ART Program, and the predicted severity of drug resistance is not sufficient. Despite these limitations, this study provides valid advice for clinical treatment, not only through survey analysis, but also based on laboratory data. The preliminary data support that the use of first-line ART regimens is effective for patients in Jiangsu. However, we should be conscious that drug resistance has been emerging. Thus, in the resource-limited settings in China with large numbers of patients, management of first-line ART and follow-up prescription guidelines is vital to prevent drug resistance from becoming uncontrollable.

## Figures and Tables

**Table 1:** Demographic characteristics of HIV-1 treated patients with virological failure on ART and univariate analyses for correlates of drug resistance.

Variables	Virologic failure		Drug resistance % (*n*)	*χ* ^2^ value	*p* value
	(*n* = 196)	%			
Age (years)				0.050	0.975
<30	38	19.4	50 (19)		
30–50	121	61.7	52.1 (63)		
>50	37	18.9	51.4 (19)		
Gender				3.424	0.064
Male	159	81.1	54.7 (87)		
Female	37	18.9	37.8 (14)		
Marital status				0.161	0.923
Single	52	26.5	53.8 (28)		
Married	110	56.1	50.9 (56)		
Other	34	17.3	50.0 (17)		
Routes of infection				4.876	0.300
Blood	11	5.6	63.6 (7)		
IDU	6	3.1	66.7 (4)		
MSM	68	34.7	57.4 (39)		
Hetro	96	49.0	43.8 (42)		
Other	15	7.7	60.0 (9)		
WHO clinical stages				10.442	0.015
I	101	51.5	40.6 (41)		
II	50	25.5	60.0 (30)		
III	28	14.3	67.9 (19)		
IV	17	8.7	64.7 (11)		
Treatment duration				18.376	0.000
<1	16	8.2	6.2 (1)		
1-2	120	61.2	52.5 (63)		
2-3	39	19.9	53.8 (21)		
>3	21	10.7	76.2 (16)		
Side effect				3.594	0.058
Yes	13	6.6	76.9 (10)		
No	183	93.4	49.7 (91)		
SMZ taken				4.280	0.039
Yes	11	5.6	81.8 (9)		
No	185	94.4	49.7 (92)		
Treatment regimen				1.909	0.592
AZT/D4T + 3TC + NVP/EFV	176	89.8	51.1 (90)		
TDF + 3TC + NVP/EFV	14	7.1	50.0 (7)		
AZT + 3TC + LPV/r	4	2.0	50.0 (2)		
AZT + TDF + LPV/r	2	1.0	100 (2)		

IDU: intravenous drug use; MSM: men who have sex with men; hetro: heterosexual; SMZ: compound sulfamethoxazole; AZT: zidovudine; TDF: tenofovir; 3TC: lamivudine; NVP: nevirapine; LPV/r: lopinavir + ritonavir.

**Table 2:** Logistic regression analysis of factors associated with HIV-1 drug resistance.

Factors	Variables	*p* value	RR	95% CI for RR	
				Lower	Upper
Gender	Male		1		
	Female	0.019	0.362	0.155	0.844

WHO clinical stages	I	0.297			
	II	0.085	1.926	0.913	4.063
	III	0.224	1.815	0.694	4.742
	IV	0.859	1.125	0.308	4.102

Treated duration	<1	0.009			
	1-2	0.014	13.616	1.715	108.109
	2-3	0.007	19.556	2.278	167.857
	>3	0.001	50.579	4.855	526.891

Side effect	Yes		1		
	No	0.186	20576	0.634	10.468

SMZ taken	Yes		1		
	No	0.123	3.707	0.700	19.633

SMZ: compound sulfamethoxazole.
